# Exploring Benefits of and Barriers to Patient Involvement Through Digital Tools in Psycho-Oncology: Qualitative Study Within the Reduct Trial

**DOI:** 10.2196/73147

**Published:** 2026-04-30

**Authors:** Caterina Schug, Alexander Bäuerle, Johanna Graf, Jana Heinen, Julia Barbara Krakowczyk, Andrea Borho, Katharina Koller, Marietta Lieb, Regina Herold, Martin Teufel, Yesim Erim

**Affiliations:** 1 Department of Psychosomatic Medicine and Psychotherapy, University Hospital Erlangen, Friedrich-Alexander-University Erlangen-Nürnberg, Schwabachanlage 6, Erlangen, 91054, Germany, 49 91318544899; 2 Comprehensive Cancer Center (CCC), University Hospital Erlangen, Erlangen, Germany; 3 Clinic for Psychosomatic Medicine and Psychotherapy, LVR-University Hospital Essen, University of Duisburg-Essen, Essen, Germany; 4 West German Tumor Center (WTZ), University Hospital Essen, Essen, Germany; 5 Center for Translational Neuro- and Behavioral Sciences (C-TNBS), University of Duisburg-Essen, Essen, Germany; 6 Department of Psychosomatic Medicine and Psychotherapy, University Hospital Tübingen, Eberhard Karls University Tübingen, Tübingen, Germany; 7 Comprehensive Cancer Center (CCC-TS), University Hospital Tübingen, Tübingen, Germany; 8 Clinic für Psychosomatic Medicine and Psychotherapy, Klinikum Stuttgart, Stuttgart, Germany; 9 Stuttgart Cancer Center (SCC), Klinikum Stuttgart, Stuttgart, Germany

**Keywords:** patient and public involvement, e-mental health, psycho-oncology, patient advisory board, digital collaboration

## Abstract

**Background:**

Patient and public involvement is essential for developing patient-centered and acceptable eHealth interventions, yet little is known about how digital collaboration with patient representatives can best be implemented in psycho-oncological research.

**Objective:**

This study aimed to identify the benefits and barriers of digital collaboration in the development of an e-mental health application and provide recommendations to optimize digital collaboration with patient representatives in psycho-oncology research.

**Methods:**

Conducted from July to September 2023, this study involved digital semistructured interviews with 5 patient representatives from the Reduct trial, a multicenter randomized controlled trial to evaluate the efficacy of the web-based psycho-oncological training Make It. The interviews were analyzed using qualitative content analysis.

**Results:**

The findings highlighted multiple advantages of digital collaboration. These included significant reductions in travel costs and effort, personal acceptance and preference for digital methods, enhanced flexibility and accessibility, a reduced health burden, increased efficiency, and scalability. Conversely, several challenges were identified: social impacts or impediments due to less face-to-face interaction, technical difficulties, compromised effectiveness and quality of communication, diverse personal preferences and acceptance levels, organizational issues, cognitive demands, socioeconomic barriers, and safety concerns. The following recommendations to optimize digital collaboration were identified: maintaining regular communication and information exchange, valuing and committing to the collaboration, using diverse communication channels, ensuring comprehensible communication, integrating feedback, fostering openness and understanding, diligent documentation and recordkeeping, and providing targeted training and support for patient representatives.

**Conclusions:**

These findings confirm and specify previously known opportunities and challenges of digital collaboration, adding crucial insights for its implementation in psycho-oncological research. This research contributes to enhancing patient-centered approaches in psycho-oncology.

## Introduction

Involving future users of an application in its development and testing is essential to ensure that the application is used and that the target group can benefit from it. In the health care sector, the users of applications are patients with various health issues. They and their health should benefit from the services developed. The concept of user-centered design (UCD) has established itself as a central element in the development of applications. UCD is an iterative design process that aims to make systems usable and useful by placing the user at the center of the development process [1]. As the central involvement of users leads to more effectiveness and better acceptance of products [2,3], patients should be involved in the development of health care applications.

Involving patients and the public, often referred to as patient and public involvement (PPI), is a process that aims to integrate the views and experiences of patients directly into the design and improvement of health care services [4,5]. Although PPI is widely discussed in health care, the exact definition and implementation are often unclear [4,6,7]. Some publications, such as the guidelines of the National Health Service of the United Kingdom, provide structures to effectively put PPI into practice [8].

The Involve briefing notes for researchers [8] serve as a guide for researchers to involve the public in public health and social research. The guidelines highlight the importance of involving patients and the public in the research process to ensure that the research is relevant, effective, and patient centered. It offers practical advice on how to conduct successful involvement, from planning and designing research to recruiting participants and disseminating research findings. The authors also highlight the positive impact that such involvement can have on the quality of research and its application in practice. The aim is to promote a research culture that recognizes the value of patient and public engagement and implements it in all phases of research. Gamble et al. [7] provide an overview of PPI in clinical trials in the United Kingdom. A German overview of the topic of PPI is provided by Schütt et al. [9].

Oncology is an area with high case numbers and a considerable mental burden for patients, which underlines the relevance of psycho-oncology in the health care system [10]. Here, eHealth applications can make a significant contribution by reaching a broad user base through their low-threshold and cost-effective services [11]. However, these applications face various barriers, such as acceptance and adherence on the part of patients [12].

PPI can be seen as an approach to address these barriers. Therefore, the Reduct trial [13], which investigated the effectiveness of the web-based psycho-oncology training Make It, was advised and supported by patient representatives from the outset. The patient advisory board of the study was included to contribute to an improved patient-friendliness of the study and training through intensive and regular cooperation [14].

Particularly in research on online care services, the question arises how digital tools can also be used for PPI (ie, patient involvement and collaboration with patients). In the era of digital technology, new opportunities are opening up for PPI in the development of eHealth services [15], including in psycho-oncology. Using digital tools to engage and collaborate with patients or patient representatives could increase the reach and efficiency of medical research [16]. Digital collaboration, a term that we use for describing the act of working together by using digital and web-based tools such as email, video calls, online meetings, and shared digital documents, offers a number of benefits, including the ability to overcome geographical and time barriers, increase accessibility, and facilitate exchanges between patients and health professionals [17], as well as between researchers and patient representatives.

However, there are also challenges and disadvantages to digital collaboration. These include possible technical problems, the risk of low acceptance of digital tools, and administrative difficulties [17]. Furthermore, the quality of communication in digital media may be reduced compared to face-to-face meetings [18].

Therefore, successful collaboration with patient representatives requires approaches that both maximize the advantages of using digital tools and minimize their disadvantages. The aim of this study was to answer the question of how and whether digital collaboration between patient representatives and researchers should be used in psycho-oncological research projects, from the perspective of patient representatives. Expert interviews were conducted and qualitatively evaluated.

## Methods

### Ethical Considerations

The Ethics Commission of the Friedrich-Alexander University Erlangen-Nürnberg (FAU) approved the study procedures (reference 21-275_3-Bn). Written informed consent was obtained from all individual participants included in the study. They were informed that their participation was voluntary and that they could withdraw at any time without giving reasons and without any other consequences. Patients also signed an informed consent form regarding publishing their data. To guarantee participant privacy and maintain strict confidentiality, all collected data were fully anonymized and deidentified before any statistical analyses were conducted. Additionally, participants received no financial or material compensation for their involvement in this research.

### Procedure

Interviews were conducted with all 5 patient representatives of the Reduct study between July 2023 and September 2023. All members of the patient advisory board were asked whether they would be willing to participate in the interviews, and all agreed.

The interviews were conducted in German and recorded as video calls using Webex (Webex by Cisco). The recorded part of the interviews lasted an average of 34 (range 26-46) minutes.

All interviews were conducted by the same research assistant, a clinical psychologist specialized in psycho-oncology (CS). The participants had already been in contact with this person prior to the interviews as part of their work on the Reduct patient advisory board. There was a predefined list of 17 questions for the semistructured interviews, which were based on the Involve guidelines. The complete list of questions can be found in Multimedia Appendix 1. The interview guide was pretested with laypersons to ensure understandability and appropriate length before being used in the study. All interviews were then transcribed verbatim so that they were available in a continuous text for the analyses.

### Sample

The sample consisted of the 5 patient representatives of the Reduct study. This was a multicenter randomized controlled trial to investigate the effectiveness of the web-based psycho-oncological training Make It. Further information on the Reduct study can be found in the work by Bäuerle et al. [13] and Krakowczyk et al. [19]. A detailed description of the recruitment of the patient advisory board and the collaboration between the patient advisory board and the research team can be found in the study by Heinen et al. [14].

Four members of the patient advisory board were female, and 1 was male. The age range of the participants was from mid-30s to mid-60s. Some of the representatives were employed, and others were retired. Concerning the level of education, all participants had completed secondary school, and most of them qualified for higher education. Their experiences with cancer had varying degrees of topicality, from 11 years in the past to rather current topicality. Four were already active at various levels in self-help groups and patient organizations before their involvement in the Reduct study.

### Qualitative Data Analysis

Each of the 5 interview transcripts was coded independently by 2 researchers (CS and A Borho). The MAXQDA software (VERBI GmbH) [20] was used for this purpose. The interviews were analyzed according to the manifest qualitative content analysis method by Mayring [21]. The following foci of interest were deductively established in a joint discussion among the researchers based on research interest: advantages and opportunities of digital collaboration between patient representatives and researchers, disadvantages and challenges of digital collaboration between patient representatives and researchers, and recommendations regarding digital collaboration with patient representatives.

Specific examples and coding rules were defined for each focus of interest. In accordance with these rules, all statements made by the interviewees that were mentioned as advantages, disadvantages, or recommendations for digital collaboration were coded and assigned to the relevant focus of interest. Selected codes from the interviews served as representative examples of each focus area. The identified statements were marked accordingly and assigned to the relevant focus areas. The 2 independent evaluators then checked the codes for consistency, discussed them, and adjusted them until a consensus was reached. All relevant statements were grouped into inductively formed thematic subgroups.

## Results

### Advantages and Opportunities of Digital Collaboration Between Patient Representatives and Researchers

In the consensus version of the codes, 43 segments were assigned to the advantages and opportunities of digital collaboration category. The codes were grouped into 6 different subcategories: reduction in travel costs and effort, personal acceptance and preference, flexibility and accessibility, reduced health burden, increased efficiency, and scalability.

“Reduction in travel costs and effort” refers to the financial savings and reduced time expenditure resulting from avoiding travel in digital collaboration. “Personal acceptance and preference” describes the individual willingness and preference of people to use digital collaboration. The “flexibility and accessibility” subcategory emphasizes the ability to participate in digital activities from any location, regardless of physical condition and other circumstances. “Reduced health burden” combines the health benefits of digital collaboration, particularly the lower physical strain and reduced risk of exposure to infectious pathogens. “Increased efficiency” describes the advantage of digital collaboration in achieving faster communication and the possibility of quicker decision-making. "Scalability" refers to the ability to implement digital communication and collaboration in larger groups and disseminate information to many participants.

An overview of the subcategories of the advantages of digital collaboration and example quotes for each is provided in Table 1.

**Table 1.** Advantages and opportunities of digital collaboration between patient representatives and researchers (N=5).

Subcategory	Quotes related to this subcategory, n	Example quote	Interviewees who mentioned an element of this subcategory, n
Reduction in travel costs and effort	11	“... because all the traveling is eliminated and it can be done much more easily.” [Interviewee 4]	3
Personal acceptance and preference	11	“I personally feel very comfortable with digital formats.” [Interviewee 4]	5
Flexibility and accessibility	10	“It’s less cumbersome now to attend appointments.” [Interviewee 1]	3
Reduced health burden	4	“... because of Covid and simply because cancer patients ... they don’t mind staying at home.” [Interviewee 2]	3
Increased efficiency	4	“You can share something, you can forward it, without, well, without it taking weeks, like it would if you were to send it by mail, for example.” [Interviewee 3]	1
Scalability	3	“We could now sit together as a group and all look at this presentation.” [Interviewee 3]	2

### Disadvantages and Challenges of Digital Collaboration Between Patient Representatives and Researchers

A total of 31 segments were assigned to the disadvantages and challenges of digital collaboration with patient representatives category in the consensus version. These could be divided into 8 different subcategories: social impact, technical challenges, effectiveness and quality of communication, personal preference and acceptance, organizational issues, cognitive requirements, socioeconomic barriers, and safety concerns.

“Social impact” refers to the loss of personal interaction and social bonding, as well as the social aspects of communication that are missing in digital form. “Technical challenges” include all aspects related to the availability and handling of the necessary technology, such as internet connection or equipment. The “effectiveness and quality of communication” category includes concerns about the efficiency and effectiveness of digital communication, including the possibility of overlooking nonverbal signals. “Personal preference and acceptance” as a challenge of digital collaboration represents individual preferences for face-to-face interactions, discomfort with using digital means, and preference for meeting in person. “Organizational issues” refer to the challenges of organizing digital meetings and the difficulty of making digital communication effective. “Cognitive requirements” describe the need for certain mental resources to be able to contribute digitally. “Socioeconomic barriers” refer to the challenges faced by people who may not have access to modern technology or are disadvantaged due to their socioeconomic situation. “Safety concerns” relate to worries about data protection, security of communication channels, and risks of cyberattacks.

An overview of the subcategories and respective example quotes of disadvantages and challenges of digital collaboration between patient representatives and researchers is provided in Table 2.

**Table 2.** Disadvantages and challenges of digital collaboration between patient representatives and researchers (N=5).

Subcategory	Quotes related to this subcategory, n	Example quote	Interviewees who mentioned an element of this subcategory, n
Social impact	7	“So, if the health insurance companies eventually decide that digital collaboration is sufficient and we no longer need to support personal interaction, we will have lost something.” [Interviewee 5]	2
Technical challenges	6	“A certain basic technical setup must be available, like an end device, a laptop for example, with a camera, perhaps not necessarily in that sense, but good audio quality, stable internet connection, yes, things like that. I would simply say there needs to be a certain basis in technical terms.” [Interviewee 1]	3
Effectiveness and quality of communication	5	“More questions are asked [during in-person events].” [Interviewee 4]	1
Personal preference and acceptance	5	“I know, however, that there are colleagues who, for whatever reasons, feel a bit uncomfortable with it.” [Interviewee 4]	3
Organizational issues	4	“For the researcher, of course, it involves a lot of organizational work.” [Interviewee 2]	2
Cognitive requirements	2	“One has to concentrate a bit.” [Interviewee 4]	2
Socioeconomic barriers	1	“So we have the combination that we know: cancer causes financial strain. But good digital collaboration also presupposes having up-to-date technology.” [Interviewee 5]	1
Safety concerns	1	“... a server that doesn’t fail or get hacked.” [Interviewee 2]	1

### Recommendations Regarding Digital Collaboration Between Patient Representatives and Researchers

In the consensus version of the qualitative analysis, 35 segments were coded for recommendations regarding digital collaboration. These were grouped into 8 subcategories: regular communication and information sharing, appreciation and commitment, diversity of communication channels, comprehensible communication, integration of feedback, openness and understanding, documentation and recordkeeping, and training and support.

The “regular communication and information sharing” subcategory contains recommendations for the regular provision of information on all steps related to the project. The “appreciation and commitment” subcategory includes recommendations for appreciative interaction and a high level of commitment on both sides (researchers and patient representatives). “Diversity of communication channels” refers to recommendations to use different communication channels to take into account the diversity of patient representatives’ needs and preferences. Offering diverse communication channels means offering different communication types or channels, for example, face-to-face meetings; telephone calls; or different web-based formats such as email, instant messaging applications, or video calls. “Comprehensible communication” describes the advocacy of measures that maximize the comprehensibility of digitally conveyed content, such as layperson-friendly language or an appropriate speed of information transfer. “Integration of feedback” includes recommendations that enable patient representatives to provide continuous feedback and make suggestions for improving collaboration, as well as their implementation. “Openness and understanding” includes recommendations that emphasize the need to promote openness and understanding on both sides (researchers and patient representatives) of each other’s concerns and worries. The “documentation and recordkeeping” subcategory includes recommendations for documenting and recording meetings, discussions, and decisions to ensure that all parties can understand what was discussed. “Training and support” refers to the provision of training materials and resources to support patient representatives in the use of digital tools and technologies.

Table 3 shows an overview of the subcategories of recommendations for digital collaboration between patient representatives and researchers, as well as example quotes of each.

**Table 3.** Recommendations regarding digital collaboration between patient representatives and researchers (N=5).

Subcategory	Quotes related to this subcategory, n	Example quote	Interviewees who mentioned an element of this subcategory, n
Regular communication and information sharing	11	“... that one remains in contact more or less constantly ... not daily, but as you did, whenever new things came up or there were questions.” [Interviewee 4]	4
Appreciation and commitment	6	“I think the conversations need to be on an equal footing.” [Interviewee 3]	3
Diversity of communication channels	3	“... This means that research budgets should also include funds for in-person meetings, so that people can see and perceive each other differently than through video calls ....” [Interviewee 5]	3
Comprehensible communication	4	“... so that the patient representatives can also understand it.” [Interviewee 3]	3
Integration of feedback	3	“We need ... a serious consideration of how the thoughts, suggestions, and ideas of the patient representatives can be incorporated in such a way that they realize: I have been listened to, or we have been listened to, and it has actually had an effect.” [Interviewee 5]	2
Openness and understanding	3	“The patient representatives, of course, must have an understanding for the researchers as well.” [Interviewee 4]	1
Documentation and recordkeeping	2	“It’s also important that the presentation, or whatever is being discussed, is sent around afterwards and, ideally, a record is provided so that everyone can follow up on what actually happened.” [Interviewee 4]	2
Training and support	2	“We need to further consider whether I can provide a device, either on loan or through health insurance or somehow else, if we were to have more digital offerings, for example.” [Interviewee 5]	2

## Discussion

### Main Findings

This interview study with all the members of the Reduct patient advisory board explored the beneficial and detrimental characteristics of digital collaboration between patient representatives and researchers in the area of eHealth research in psycho-oncology. It also explored recommendations that should be taken into account.

In line with previous literature, a reduction in travel costs and expenses, flexibility and accessibility, increased efficiency, and scalability were identified as advantages of digital collaboration [17]. Thus, these advantages were confirmed for the specific context of collaboration between patient representatives and researchers in the field of psycho-oncology. In addition, the reduced health burden of digital forms of collaboration was highlighted as an advantage. This appears to be particularly relevant in pandemic and postpandemic times and when working with groups of people with health problems.

Personal acceptance and preference was identified as an issue to be considered with regard to both the advantages and disadvantages or challenges of digital collaboration, which underlines the need to focus on the needs and personal preferences of the respective users also in terms of digital PPI. Therefore, UCD [1] and related concepts already appear to play a role in the design of the collaboration between the people (groups) involved and not only in the development of an application.

The disadvantages and challenges subcategories of “social impact,” “technical challenges,” “effectiveness and quality of communication,” and “security concerns” are also in line with the previous literature on digital collaboration [17,18]. Therefore, these aspects could also be confirmed for the practical context of PPI in the field of psycho-oncology. The identified category of cognitive requirements should be taken into account, particularly for the health care sector and when working with patient representatives who were or are themselves affected by an illness, as digital participation requires health and mental resources. Socioeconomic barriers also appear to be relevant for collaboration with patient representatives in research.

With regard to the recommendations on digital collaboration, the results are very much in line with the National Health Service [8] recommendations on PPI in general, particularly in terms of regular communication and information sharing, clear communication, documentation and recordkeeping, and the integration of feedback. These aspects appear to be particularly relevant in the digital format and should be taken into account in digital collaboration. It should also be emphasized that appreciation and commitment as well as openness and understanding were identified as recommendations, which underlines the importance of social skills and resources, especially in digital collaboration. The finding that a variety of communication channels is recommended needs to be highlighted for the context of psycho-oncology research with patient representatives: using hybrid models that combine digital with face-to-face elements might ensure broad participation and deeper human interaction. The finding that training and support are considered advisable underlines the importance of creating training and support structures for patient representatives that facilitate the use of digital tools. In line with previous research on PPI [22,23], we would like to share the experiences and insights gained in the context of the Reduct trial to facilitate and inspire PPI in future research projects.

### Strengths and Limitations

One of the strengths of this study is that we were able to address this highly relevant practical topic with the help of experts as interview partners who had many years of experience in supporting web-based psycho-oncological training and working with researchers in this field. Therefore, the basis of the analyses was a wealth of experience gained over many years as patient representatives in the field of PPI in the psycho-oncological health care system and in the digital field.

A methodological strength of this study is that the analyses were carried out by 2 independent coders to increase the objectivity of the results. However, it needs to be mentioned that the interviewer and interviewees knew each other and worked together before the interviews, which may have impacted the answers and introduces potential social desirability or response bias.

It should be noted that some of the recommendations identified for digital collaboration are also applicable to collaboration in general; nevertheless, they were mentioned in a digital context.

The fact that the gender distribution between men (20%) and women (80%) was not balanced could also be seen as a limitation. For this reason, the generalizability of the results should be assumed with caution.

The sample size of 5 interviewees can be mentioned as a limitation regarding diversity, saturation, or transferability of findings. Nevertheless, these were all official members of the patient advisory board of the Reduct trial who were participating for the same amount of time and shared expertise on this trial.

Another limitation of this study is the high level of education of the sample, which could possibly limit its representativeness. However, a strength is that the interviewed sample was heterogeneous in terms of age, profession, and experience in PPI or self-help.

### Conclusions

The results of this interview study suggest that digital collaboration between patient representatives and researchers in the field of psycho-oncology is advantageous in terms of reducing travel costs and effort, flexibility and accessibility, health relief, efficiency enhancement, and scalability. Disadvantages of the digital form of collaboration in this context may include social impacts, technical challenges, reduced effectiveness and quality of communication, increased organizational issues, cognitive demands, socioeconomic barriers, and security concerns. The personal preference of each participant also seems to play a role in the perception of digital collaboration. Recommendations for digital collaboration include regular communication and information exchange between patient representatives and researchers, appreciation and commitment, a diversity of communication channels, understandable communication, integration of feedback, openness and understanding, documentation and recording of digital collaboration content, and training and support for patient representatives regarding technical issues. In the psycho-oncological context, the recommendation to ensure comprehensible communication reflects the need to convey complex and often emotionally charged information in a clear and accessible manner. Layperson-friendly language and an appropriate speed of information transfer are essential to adequately understand clinical processes and meaningfully contribute to collaborative decision-making. Similarly, the call for openness and understanding highlights the importance of acknowledging the differing perspectives and expectations of researchers and patient representatives. Mutual insight into each other’s concerns and working contexts fosters trust and supports constructive collaboration within the sensitive setting of psycho-oncology.

Given the high relevance of PPI in the field of psycho-oncology, it seems beneficial for research projects to strongly consider collaboration with patient representatives via digital tools and take into account the benefits and challenges and ways to address them in the specific context of each patient group.

## Supplementary material

10.2196/73147 Multimedia Appendix 1 Interview Questions

## Abbreviations

PPI: patient and public involvement
UCD: user-centered design
