# Supplementary material for: Exploring Benefits of and Barriers to Patient Involvement Through Digital Tools in Psycho-Oncology: Qualitative Study Within the Reduct Trial
Source: JMIR Form Res. 2026 Apr 30;10:e73147. doi: 10.2196/73147 (PMC13132482; doi:10.2196/73147)
Supplement: Multimedia Appendix 1 [file formative-v10-e73147-s001.docx]

Appendix 1

**Interview questions**

1. It is very important to include patients' views and experiences in research. The following section will focus on research into online psychological support services for cancer patients.

In your opinion, what is the aim of involving patients in research on such services?

2. What motivates you to serve on the patient advisory board of the Reduct study?

3. What qualities do you think patient representatives should have?

4. How much scientific knowledge do you think patient representatives need to participate in a research project on online support services?

5. How did you come to join the patient advisory board of the Reduct study?

6. Were you already active in the field of patient welfare, for example in self-help groups?

7. How do you think researchers can best reach interested patient representatives about online support services?

8. What conditions do you think need to be in place to enable good collaboration between researchers and patient advocates?

9. Do you think digital collaboration (e.g. via email and video calls) is appropriate?

10. What opportunities do you think digital collaboration offers?

11. What challenges do you think digital collaboration poses?

12. What do you hope to gain personally from your work as a patient representative?

13. How should researchers enter into dialogue with patients about online support services?

14. How do you recognise successful inclusion of the patient's perspective in a research project on an online support service?

15. What should be considered in the project management and planning of an online support programme in order to include patient representatives?

16. In which phase of a research project (brainstorming, preparation, implementation, evaluation) do you find the involvement of patient representatives particularly important? Why?

17. How do you think the patient perspective can best be included in all phases?
